# Synergistic Effect of Diallyl Sulfide With Zinc Oxide Nanorods: A Novel and Effective Approach for Treatment of Acute Dermatitis in Model Animals

**DOI:** 10.3389/fmicb.2018.00586

**Published:** 2018-04-18

**Authors:** Mohd A. Rauf, Swaleha Zubair, Hira Ateeq, Khadija Dabeer, Subodh Pachauri, Mohd Ajmal, Mohammad Owais

**Affiliations:** ^1^Interdisciplinary Biotechnology Unit, Aligarh Muslim University, Aligarh, India; ^2^Department of Computer Science, Aligarh Muslim University, Aligarh, India; ^3^Department of Biotechnology, NMAM Institute of Technology, Nitte, India; ^4^Department of Anatomy, Jawaharlal Nehru Medical College, Aligarh Muslim University, Aligarh, India

**Keywords:** methicillin resistant *Staphylococcus aureus* (MRSA), antibacterial, antibiofilm, ZnO-NRs, diallyl sulphide (DAS), skin infection

## Abstract

Besides inciting persistent and recurrent nosocomial afflictions, *Staphylococcus aureus* (*S*. *aureus*), a biofilm forming pathogen, poses an increased risk of several skin as well as respiratory tract infections as well. Emerging antimicrobial resistance trend asks to search for an alternate non-antibiotic based option to combat *S. aureus* pathogen. In the present study, we evaluated synergistic antimicrobial potential of Zinc oxide nanorods (ZnO-NRs) and diallyl sulphide (DAS) emulsion against methicillin resistant *Staphylococcus aureus* (MRSA). The antimicrobial assessment study suggests that the ZnO-NR and DAS emulsion effectively suppressed both sensitive *S. aureus* as well as MRSA isolates. The combination treatment showed enhanced activity even at a lower concentration as compared to the single treatment based on ZnO-NRs and DAS emulsion alone. The ZnO-NRs-DAS combination showed significant inhibition of MRSA biofilm as well. The data suggest that a combination therapy, comprising of ZnO-NRs and DAS emulsion, successfully treated experimental dermatitis infection caused by MRSA in mice model.

## Introduction

*Staphylococcus aureus, a* Gram-positive commensal bacterium, causes nosocomial and community associated infections worldwide (Zetola et al., 2005). In general, *S. aureus* induces mastitis and several moderately severe infections of the skin or respiratory tract. Besides, it may also cause life threatening perilous forms of diseases such as necrotizing fasciitis or necrotizing pneumonia etc. (Otto, 2010, 2012; Zazo et al., 2016). The emerging spread of antibiotic resistance in microorganisms and the failure of available drug regimen to treat them, poses a major threat to human health (Hay and Morris Jones, 2010; Khan and Khan, 2016). For example, emergence of methicillin resistance in *S. aureus* is one of the most critical therapeutic issues. It was assessed that globally 33% individuals have *S. aureus* on their bodies at any given time, fundamentally in the nose and also on the skin. As indicated by CDC, Atlanta, around 80,461 intrusive MRSA contaminations and 11,285 death cases had been reported in 2011^1^ (Harvard Health Blog, 2016).

Bio films are structured multi-cellular communities that irreversibly adheres to each other in a self-produced extracellular matrix (Costerton et al., 1995; Doulgeraki et al., 2017). Several environmental factors, such as nutrient and oxygen availability led to the formation of bacterial biofilm (Okeke et al., 2005). Biofilm plays crucial role in imparting high resistance to conventional antimicrobial agents (Mah and O’Toole, 2001; Sugimoto et al., 2018). Besides restricting penetration of antimicrobial agents, biofilm does not allow entry of nutrients to bacterial niche that eventually ensues in slow growth. *S. aureus* has also been reported to attain increased resistance to a number of antimicrobial drugs through biofilm formation (Donlan and Costerton, 2002; Hall-Stoodley et al., 2004; Gordon and Lowy, 2008; Otto, 2008; Foster, 2017).

Herbs and plants in general have been used in traditional medicine to cure a number of ailments since ancient times. For example; garlic has been traditionally used in routine culinary as well as use as medicine in many parts of the world (Cowan, 1999; Hoareau and DaSilva, 1999). Among various components, allyl sulfide derivatives have been reported to possess strong antimicrobial activity. For example, the allyl sulfide analogs of the garlic oil such as DAS and DADS exhibited concentration-dependent microbicidal activity against *H. pylori*, MRSA (Ross et al., 2001; Shah, 2005), *E. coli* and *Candida* sps etc. (Alam et al., 2009, 2013).

Antimicrobial nanoparticles and nano carriers have potential to treat infectious diseases caused by both drug resistant as well as susceptible bacteria (Sondi and Salopek-Sondi, 2004; Rai et al., 2012). Drug bearing nanoparticles, release desired amount of their payload at ailment site and successfully eradicate bacterial infection. Of late, there has been an escalating enthusiasm to decipher the antimicrobial properties of designer nano-scale metal particles (Singh, 2010; Sotiriou and Pratsinis, 2010). Among various metals, ZnO nano-particles, have found wide applications in antimicrobial therapy, disease treatment and food packaging etc. (Riggio et al., 2010; Zhang and Xiong, 2015). ZnO nano-particles with potential antimicrobial activities have been listed safe by the FDA, United States (Food and Drug administration 21CFR182.8991). Both anticancer as well as bacterial properties of ZnO-NPs can be correlated with their unique ability to generate reactive oxygen species (ROS).

To combat methicillin resistant *S. aureus* (MRSA), the present study envisages a ZnO-NRs and DAS based combination therapy. We systematically evaluated effectiveness of ZnO-NRs-DAS combination to treat MRSA infections. Finally, we evaluated the novel combination therapy comprising of ZnO-NRs and DAS bearing emulsion formulation to cure MRSA induced skin infection in Balb/c mice (**Figure 10**).

## Materials and Methods

### Materials

Luria Broth (LB) and agar powder were procured from Hi-media (India). Zinc acetate powder was purchased from Merck research laboratories Private limited (INDIA). Diallyl sulfide (DAS 50%), were purchased from Sigma-Aldrich, United States. The two strains viz. MRSA (ATCC BAA-1708) and MRSA (ATCC 43300) were a kind gift from Dr. M. Shahid the Chairperson, Department of Microbiology, Jawaharlal Nehru Medical College, AMU, Aligarh, India. The standard strains were sub-cultured in LB. The cultures were stored at -20°C in 20% glycerol, for long term preservation. All experiments were performed with the freshly grown cultures. All other chemicals and solvents used were of analytical grade and acquired locally.

### Synthesis of ZnO Nanorods

#### Bio-Fabrication of ZnO Nanorods Employing Hen Egg White (ZnO-NRs)

We followed published procedure using hen egg white to fabricate ZnO-NRs (Shoeb et al., 2013; Khan et al., 2015). Briefly, freshly extracted egg white was isolated from hen egg (purchased from local supermarket). A fixed volume (30 ml) of egg white was first mixed with 15 ml of deionized water under vigorous stirring at ambient temperature (25°C) until a homogeneous solution was obtained. Zinc acetate (100 mM) was added slowly to the egg white under vigorous stirring at 25°C. The mixture was further stirred for 3 h to obtain a homogeneous solution. An aliquot (20 ml) was transferred to a 30 ml microwave glass tube (tightened with septum). The sample was exposed to the microwave (Anton paar monowave 300), for 60 min at 200°C. Microwave reactor was cooled down to 40°C to obtain a milky white precipitate solution. The synthesized precipitate was centrifuged at 20,000 × *g* and washed extensively with water, followed by vacuum drying at 80°C for 6 h to obtain ZnO-NRs.

#### Characterization of As-Synthesized ZnO-NRs

The synthesized ZnO-NRs were characterized employing various spectroscopic techniques viz UV-Visible spectroscopy, Electron Microscopy, X-ray diffraction and dynamic light scattering (DLS). The information, regarding detailed methodology used, is provided in the Supplementary Section.

#### Effect of Nano-Formulations on Bacterial Growth

Employing standard antimicrobial procedures minimum inhibitory concentration (MIC) of ZnO-NRs was determined against MRSA strains. Detailed methodology is provided in Supplementary Section.

#### Synergistic Effect of as Synthesized ZnO-NRs With Diallyl Sulfide (DAS) as Determined by the Disk-Diffusion Method

After establishing antimicrobial potential of as-synthesized ZnO-NR and DAS emulsion based therapy against MRSA at individual level, the synergistic effects of the two formulations against MRSA ATCC 43300 and BAA-1708 strains was determined. Sterile disks were impregnated with ZnO-NRs and DAS emulsion and placed on bacteria harboring plates followed by incubation at 37°C for 24 h. Antibacterial activity was expressed in terms of diameter of the zone of inhibition, measured in millimeters. The assays were performed in triplicate (Wiegand et al., 2008; Rauf et al., 2017).

#### Intracellular ROS Production by As-Synthesized ZnO-NRs

ZnO-NRs mediated generation of intracellular ROS in treated bacterial cells was measured employing fluorescent probe 2,7-dichloro-fluorescein diacetate (DCFH-DA) (Eruslanov and Kusmartsev, 2010). The DCFH-DA passively diffuses across the bacterial cell membrane. Once internalized it gets deactivated by esterases to form a non-fluorescent 2,7-dichlorofluor-escein (DCFH). The DCFH dye reacts with generated ROS to produce the fluorescent product 2,7-dichlorofluorescein (DCF) which gets trapped within the cell making it fluorescent. Briefly, the freshly cultured bacteria (MRSA ATCC BAA 1708 ∼ 10^6^cfu/ml) were washed three times with fresh medium. DCFH-DA was mixed with the culture and incubated in shaking incubator for 30 min at 37°C. The cells were pelleted down and washed to remove the unbound DCFH. The experimental and control cells were used for FACS analysis and also visualized under a fluorescence microscope (Zeiss model, United States). Keeping into account the fact that the fluorescence intensity is proportional to the quantity of ROS produced, the cells were analyzed using cytometric acquisition employing BD FACS ARIA II Flow Cytometer and results were analyzed using FACS DIVA^®^ analysis software.

#### MRSA Viability Assay Using SYTO 9 and Propidium Iodide (PI) Staining

To assess the viability of MRSA ATCC BAA 1708 following treatment with ZnO-NRs and DAS emulsion, a SYTO9-PI bacterial viability kit (Invitrogen, CA, United States) was used. An aliquot (10 μl) consisting of an equal proportion of SYTO9 and PI mixture was added to the treated cells in the 6 well-plates and incubated for 15 min in the dark at 25°C. After the stipulated incubation time, the cells were washed with cold sterile PBS and mounted on glass slide. The cells were observed under fluorescence microscope (Zeiss, United States) (Miller and Quarles, 1990; Rauf et al., 2017).

#### XTT Biofilm Assay

The XTT based biofilm assay was employed to assess the antibiofilm activity of as-synthesized NRs (Xu et al., 2016). Briefly, post mature biofilm formation; the wells of the plate were carefully rinsed with sterile PBS to remove non adherent cells. The mature biofilm was treated with increasing concentration of various ZnO-NRs-DAS based formulations and incubated at 37°C for 24 h. After stipulated incubation period, 2,3-Bis(2-methoxy-4-nitro-5-sulfophenyl)-5-[(phenylamino) carbonyl]-2H-tetrazolium hydroxide (XTT) solution in PBS, was added at a final concentration of 5 mg/ml. The obtained solution was filter sterilized using a 0.22-mm pore-size filter and stored at -80°C until required. Menadione solution (0.4 mM) was prepared and filtered immediately just before the commencement of each assay. Adherent cells were washed with PBS and exposed to XTT solution followed by addition of 2 μl menadione. The solution was transferred to a new plate after incubation in the dark for 4 h at 37°C and scanned using a microtiter plate reader (BIO-RAD Microplate reader at 490 nm). Experiments were performed in triplicate. The data are expressed as means ± SD.

#### Ultrastructure Analysis of ZnO-NR-DAS Treated Bacterial Cells Employing Electron Microscopy

Methicillin resistant *Staphylococcus aureus* isolate was exposed to various ZnO-NRs formulations for 60 min, at 37°C, with constant agitation (at 250 rpm). The cell suspension was washed five times in modified-TSB medium and three times in PBS to remove unbound or loosely associated nanoparticles. The cells (approximately 10^8^ CFU) that interacted with ZnO-NRs formulations at stipulated time interval, were prepared and imaged using SEM. Additionally, the bacteria were imaged by transmission electron microscopy (TEM) as well. Briefly, the bacteria, (∼ 10^9^ CFU) were fixed with 1% glutaraldehyde in PBS and subsequently exposed to 1% osmium tetroxide in water, for 24 h each. The sample was transferred onto a 0.22 μm pore size filter (Millipore) and substituted with acetone, and subsequently with liquid CO_2_ in a critical point drying apparatus (SPI, United States). Filter paper sections were metallised with gold, by sputter coating, and imaged with a JEOL JSM-6390 LV (Hartmann et al., 2010).

#### SDS–PAGE of ZnO-NR-DAS Treated MRSA Whole Cell Lysate

It can be argued that treatment with ZnO-NRs formulation will cause degradation of various structural as well as enzymatically active proteins of MRSA. The proposed degradation of MRSA associated proteins was ascertained by SDS–PAGE analysis (Guo et al., 2016). The ZnO-NR treated bacterial cells were washed twice with phosphate buffer (5 mM, pH 7.2) and the proteins were isolated with the help of Invitrogen kit. The protein content was estimated employing BCA protein assay kit. The protein samples belonging to various treated groups were loaded on SDS–PAGE gel. The SDS–PAGE was performed with a 5% stacking gel and a 12% separating gel. The gel was stained with CBB.

#### Antimicrobial Potential of ZnO-NRs-DAS Emulsion Against Experimental MRSA Skin Infection

Six to eight week old male BALB/c mice (5 mice in each group) were divided in five different groups. Animals from each group were anesthetized by intra-peritoneal (ip) injection of a ketamine-xylazine cocktail and subsequently shaved on the dorsal surface. The skin of mouse was rasped with sterile scalpel blades to develop bruised surface. The surface of each wound was inoculated with 50 μl of the bacterial culture (10^8^ CFU of MRSA BAA 1708 strain). The infection was allowed to establish for 72 h. The treatment schedule was followed as:

(1) Group 1: Healthy mice treated with normal saline only.
(2) Group 2: Mice infected with MRSA followed by treatment with normal saline only.
(3) Group 3: Mice exposed to MRSA followed by treatment with ZnO- NRs (1 g/kg body weight).
(4) Group 4: Mice exposed to MRSA followed by treatment with DAS (0.5 g/kg body weight).
(5) Group 5: Mice exposed to MRSA followed by treatment with ZnO-NRs and DAS emulsion (1:1 ratio) at the dose of 0.3 g/kg body weight.

Prior to application of the various nano-formulations, each mouse was fitted with an Elizabethan collar (Braintree Scientific, Braintree, MA, United States) to prevent removal and ingestion of applied formulation, following the protocol of Mortensen et al. (2008), Pati et al. (2014). The specific group of animals was treated with corresponding formulation as specified above on day 4 and day 7 post exposure to the infection. Ten days post infection, anesthetized mice were sacrificed. Infected skin sample was homogenized using a tissue homogenizer (Silverson Machines, East Longmeadow, MA, United States) and the residual MRSA burden was determined by plating serially diluted homogenized samples on TSB agar plates.

For histopathological studies, infected skin tissues was sliced (from mice belonging to various groups) and fixed in 10% formaldehyde solution, dehydrated in ascending grades of ethyl alcohol, cleared in xylol and mounted in molten paraplast at 58–62°C. The finely cut thin section was stained with hematoxylin and eosin stain and evaluated for any morphological changes under an Olympus BX40 microscope (PA, United States) for the comparative study (Kugelberg et al., 2005; Rauf et al., 2017).

#### Ethical Clearance of the Study

Inbred BALB/c mice (6–8 weeks old, 20 ± 2 g) were obtained from the Institute’s Animal House Facility. The BALB/c mice were housed in commercially available polypropylene cages and maintained under controlled temperature conditions on a 12hr light-dark cycle and had free access to food and water *ad libitum*. All the animal experiments were performed according to the National Regulatory Guidelines issued by the Committee for the Purpose of Control and Supervision of Experiments on Animals (CPCSEA). Our approval ID was *332/CPCSEA.*

## Results and Discussion

### Fabrication of ZnO-NRs Employing Egg White as Synthesis Template

Egg white, primarily consisting of polypeptide chains with hydrophilic –NH_2_, -O-, -–NH-C=O-O and -COOH functional groups, has been reported to serve as a bio template facilitating nanoparticle formation (Shoeb et al., 2013; Khan et al., 2015). While working as a biotemplate, egg white facilitates embedding of Zn(OH)_4_^2-^ in its long chain orientation. Under the reaction conditions, numerous Zn(OH)_4_^2-^ seeds impregnate the egg white gel matrix. Once the reaction proceeds, Zn(OH)_4_^2-^ crystal gradually mature leading to formation of a rod like structure across the egg white matrix and eventually rod shaped ZnO-nano-particles were synthesized.

$$\begin{array}{c} \;\begin{array}{c} \mathrm{Egg}\;\mathrm{White}\;({-NH}_{2}\;\mathrm{from}\;\mathrm{ovalbumin})\;+\;H_{2}O\;\to \;\mathrm{Egg}\;\mathrm{White}({-NH}_{2}{}^{+})\;+\;{\mathrm{OH}}^{-} \end{array} \end{array}$$

$$\begin{array}{c} {\mathrm{Zn}}^{2}{}^{+}\;+\;{2OH}^{-}\;\to \;\mathrm{Zn}\;{\left(\mathrm{OH}\right)}_{2}\;\to \;ZnO - NRs\;+\;H_{2}O \end{array}$$

### Characterization of As-Synthesized ZnO-NRs

**FIGURE 1:**
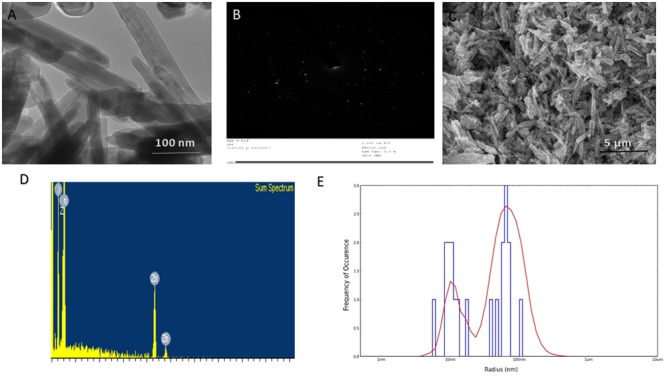
Size and shape analysis of as-synthesized ZnO-NRs. **(A)** TEM analysis depicting shape and size of the as-synthesized ZnO-NRs. **(B)** Crystalline diffraction pattern of as-synthesized ZnO-NR as revealed by TEM analysis **(C)** SEM micrograph depicting microstructure and surface topology of as-synthesized ZnO-NRs. The inset shows SEM image of the ZnO-NRs at higher magnification **(D)** EDAX-spectrum showing elemental analysis of as-synthesized ZnO-NRs. **(E)** Size analysis-of as-synthesized ZnO-NRs as ascertained by DLS analysis.

**FIGURE 2:**
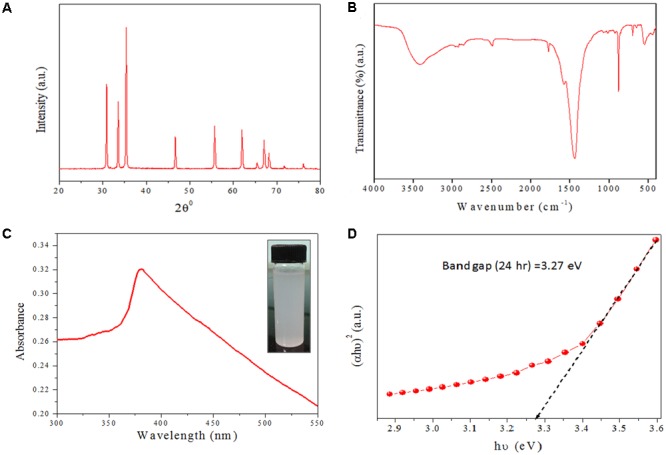
Structural characterizations of as-synthesized ZnO-NRs **(A)** XRD pattern of as-synthesized ZnO-NRs was recorded with the 2Ø angle in range of 20–80. XRD pattern of NRs showed well-resolved diffraction peaks of the crystalline structure. **(B)** Representative FTIR spectrum of the as-synthesized ZnO-NRs. **(C)** UV–visible absorption spectrum of as-synthesized ZnO-NRs fabricated by employing egg white as bio template. **(D)** Tauc plot representing the energy band gap of as-synthesized ZnO-NRs.

The as-synthesized ZnO-NRs were analyzed for their size and surface characteristics. **Figure 2C** corresponds to the scanning electron micrograph of as-synthesized ZnO-NRs. The film deposited on a carbon tape clearly demonstrates the formation of secondary ZnO-NRs (average size 50 nm). The elemental analysis employing EDAX indicates predominant presence of zinc and oxygen element in as-synthesized ZnO-NRs (**Figure 2D**). Moreover, the EDAX mapping analysis suggests no other elemental contamination impurities present in the as-synthesized ZnO-NRs (**Figure 2D**). The TEM image suggests that ZnO-NRs to be actually composed of several particles of varying dimensions grouped in clusters (**Figure 2A**). ZnO-NRs acquire a rod like morphology with a size range of 10–60 nm. The electron diffraction image **Figure 2B** indicate the presence of metallic zinc in the synthesized NRs. In general, diameter determined by DLS measures larger (∼90 nm) as compared to the size-dimensions determined by TEM analysis (**Figure 1E**). In fact, different methods of size-determination can produce conflicting data. TEM provides information about the size and shape of individual nanoparticles dried under high vacuum. dynamic light scattering (DLS) measures diffusion in particle dispersions, which can be interpreted to yield an ensemble average hydrodynamic particle diameter using the Stokes-Einstein equation. This causes a discrepancy between TEM and DLS based size determination as the former corresponds to dried form while hydrodynamic and electro kinetic parameters are operative in DLS measurement. (Ito et al., 2004; Shoeb et al., 2013; Khan et al., 2015; Lee et al., 2017).

### XRD Analysis of As-Synthesized ZnO-NRs

XRD data analysis of as-synthesized ZnO-NRs shows 11 peaks obtained at 2𝜃 = 31.01°, 34.21°, 35.64°, 47.10°, 56.02°, 62.15°, 65.68°, 67.51°, 69.01°, 72.08°, and 76.24° which correspond to the crystal planes [100], [002], [100], [102], [110], [103], [200], [112], [201], [004], and [202] of polycrystalline wurtzite structure with JCPDS 5-0664and space group P63mc (Lee et al., 2017), respectively. **Figure 1A** represents the crystal structure of ZnO-NRs as characterized by X-ray diffraction analysis. A peculiar line broadening signal of the XRD peaks reveals that the synthesis material consists of crystalline natured particles with miniscule range. The XRD data suggests the absence of any impurity, with the average crystalline particle size ∼42 nm, as calculated using Debye–Scherrer formula.

### Identification of Functional Groups Present in As-Synthesized ZnO-NRs Employing FTIR Spectroscopy

In order to identify the functional group characteristics of as-synthesized ZnO-NRs, Fourier transform infrared (FTIR) spectroscopy, was conducted (**Figure 1B**). The wide-ranging elevation in the peak lying in the range of 3500–3300 cm^-1^ corresponds to the OH stretching. The peak at 1585 cm^-1^ corresponds to an OH bending vibration (Shoeb et al., 2013; Khan et al., 2015). The minuscule peaks at 830 cm^-1^ and 535 cm^-1^ clearly suggests Zn–O stretching unlike the peaks at 1585 cm^-1^ and 1030 cm^-1^ that can be assigned to the C–O stretching vibration and unreacted zinc precursor left after the reaction. The peaks at 630 cm^-1^ and 830 cm^-1^ correspond to Zn–O stretching and confirms fabrication of ZnO-NRs.

### Optical Properties of As-Synthesized ZnO-NRs

The optical properties of as-synthesized ZnO-NRs were assessed employing the UV-VIS spectroscopy as a function of wavelength (Guo et al., 2009). Electronic status was studied on the basis of energy band gap (Eg), which is an estimate of the energy difference between the valance band (Ev) and the conduction band (Ec) (Shoeb et al., 2013; Khan et al., 2015), Because of their electronic structure as well as redox potentials, metal oxide generate the specific type of ROS like OH, 1O^2^, or O^2-^during specific conditions. ZnO-NRs mediated ROS generation result in their observed antimicrobial or anticancer activity. In accordance to the previous studies, the UV-Visible spectrum analysis shows a distinctive absorption peak corresponding to the wavelength of 370 nm (**Figure 1C**) (Shoeb et al., 2013; Khan et al., 2015; Vijayakumar et al., 2015). The intense absorption peak of ZnO-NRs revealed the narrow size particle distribution in the nano regime. Additionally, absorption peak can be designated to the transition of valance band of the conduction band (O2p → Zn3d) (**Figure 1D**).

### Anti-Bacterial Activity of As-Synthesized ZnO-NRs and DAS Emulsion Combination

**FIGURE 3:**
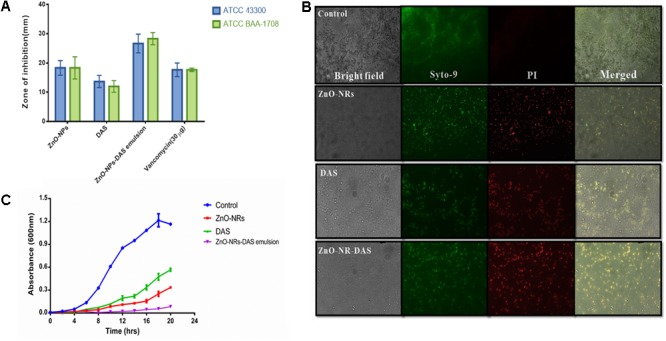
**(A)** The antibacterial potential of ZnO-NR-DAS combination therapy against MRSA as ascertained by measuring zone of inhibition incurred. **(B)** Fluorescence microscopic images corresponding to live dead cell assay employing PI and Syto-9 dyes to ascertain anti-bacterial potential of ZnO-NRs against MRSA ATCC BAA-1708 cells. **(C)** Growth kinetics analysis of MRSA upon their treatment with ZnO-NR-DAS.

The antimicrobial action of as-synthesized ZnO-NRs was evaluated against MRSA strains. Standard antibiotic vancomycin was taken as a control. The MIC values of the synthesized nanorods were found out to be 64 μg/ml for MRSA ATCC 43300 and 64 μg/ml for MRSA ATCC BAA-1708. The DAS (50%) alone had an MIC of 128 μg/ml for both the strains. Interestingly, the emulsion of ZnO-NRs-DAS had an MIC of 16 and 8 μg/ml, respectively. Next, agar diffusion assay was performed to further confirm the observed bacterial inhibition properties of ZnO-NRs-DAS (**Figure 3A**). The zone of inhibition assay suggests that the combination of ZnO-NRs and DAS emulsion possesses effective bactericidal activity against the two tested MRSA strains.

To further assess antimicrobial property of ZnO-NRs and DAS emulsion combination, we performed live dead assay employing confocal microscopy. Bacterial cells from log phase were exposed to various ZnO-NRs formulations for 3 h followed by staining with the PI and Syto-9 dyes. The dye, PI, penetrates cells with severed membrane lesions in dead cells. The PI uptake data had been depicted in **Figure 3B**. The dead cells appear as sharp red dots in various treated group as the dye binds itself with DNA of killed cells (dead cells). The observed PI binding to the double stranded DNA indirectly suggests that the synthesized compounds damage the bacterial cell wall (Kulshrestha et al., 2014; Ahmad et al., 2015; Khan et al., 2017) [Data not shown for MRSA 43300].

Agar diffusion method was also used to further establish anti-bacterial potential of ZnO-NRs and DAS emulsion combination. The as-synthesized ZnO-NRs showed promising antibacterial effect against both the MRSA strains as revealed by agar diffusion method (Espitia et al., 2012; Vijayakumar et al., 2015) (**Table 1**). The supplementation of ZnO-NRs with DAS had more potent antibacterial effects as compared to the ZnO-NRs alone. The observed effect can be explained on the premise that DAS itself suppresses the growth of the bacteria and supplement antibacterial activity of zinc oxide nanorods. The growth curves of bacterial cells treated with ZnO-NRs-DAS emulsion indicated that the combination therapy could significantly inhibit the growth and reproduction of bacterial cells as compared to the individual treatment module with DAS and ZnO-NRs alone (**Figure 3C**).

**Table 1:** Antibacterial potential of ZnO-NR-DAS combination against MRSA Strains.

Strains				
S.No.	ZnO-NRs	DAS	ZnONR-DAS emulsion	Van (30 μg/disk)
ATCC 43300	18.33 ± 2.516	13.667 ± 2.082	26.667 ± 3.214	17.67 ± 2.309
ATCC BAA-1708	18.33 ± 3.7	12 ± 2	24.4 ± 2.082	17.67 ± 2.5

**FIGURE 4:**
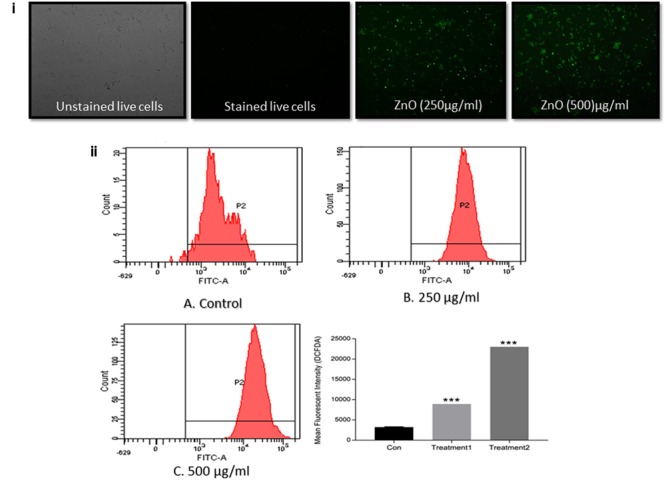
ZnO-NR mediated ROS production in MRSA ATCC BAA 1708 **(i)** ROS generation in bacteria upon treatment with ZnO-NRs as depicted by Fluorescence microscopy. **(ii)** Flow cytometric analysis corresponding to total ROS generation as revealed by DCFDA dye accumulation. The bacterial were treated with 20 μM DCFDA for 30 min at 37°C. Flow cytometric histograms represent fluorescence intensities acquired in 10,000 events in control, MRSA ATCC 43300 cells treated with ZnO-NRs for 24 h, respectively, using £x/£m = 495/529 nm. ^∗∗∗^*P* ≤ 0.001.

**FIGURE 5:**
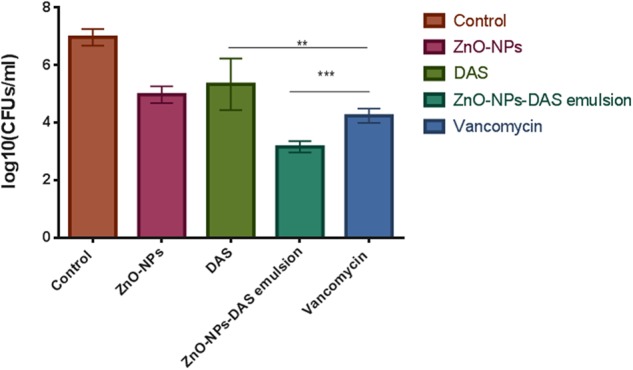
Antibacterial potential of ZnO-NR-DAS against sensitive as well as methicillin resistant *S. aureus*. The residual MRSA ATCC BAA 1708 surviving after exposure to various forms of ZnO-NR-DAS combination depicted as colony forming unit. ^∗∗^*P* ≤ 0.01, ^∗∗∗^*P* ≤ 0.001.

The ZnO-NRs mediated ROS production was assessed using the fluorescence dye, DCFHDA. In general, ZnO-NRs induce the generation of ROS that causes alteration and decrementation of cellular proteins, DNA, and lipids, which eventually lead to cell death (Akhtar et al., 2012; Rauf et al., 2017). As shown in **Figure 4i**, a significant increase in DCF fluorescence was observed upon the treatment of bacteria (MRSA ATCC BAA-1708) with ZnO-NRs. Further, FACS analysis of the sample suggested augmentation in the ROS production after exposure to ZnO-NRs (**Figure 4ii**) **[**Data not shown for MRSA ATCC 43300]. A significant decrease in bacterial burden [*P*-value < 0.01 (^∗∗^)] was observed against both the MRSA strains on treatment with ZnO-NRs-DAS combination as compared to vancomycin (positive control) treatment (**Figure 5**). Interestingly, ZnO-NRs treatment was found to be equally effective to that of Vancomycin mediated killing of MRSA (**Figure 4** and **Table 2**). [MRSA ATCC 43300 responded in same manner upon treatment with ZnO-NRs data not shown].

**Table 2:** *In vitro* CFU counts depicted as log10 cfu/ml.

Pathogen	Groups	Log_10_ CFU/mL
MRSA 43300	Positive control	7.1735
	ZnO-NRs	4.90
	DAS	5.370
	ZnO NR-DAS emulsion	3.30
	Vancomycin	4.17

### Antibiofilm Activity of As-Synthesized ZnO-NRs-DAS Combination

**FIGURE 6:**
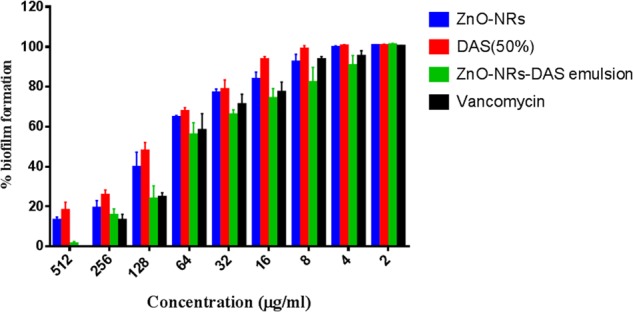
Antibiofilm activity of various ZnO-NR-DAS combination. Effect of ZnO-NR-DAS combination against MRSA ATCC BAA-1708 biofilm. Growth inhibition was assessed by comparing relative metabolic activity (RMA) determined employing XTT assay; untreated control was considered to show100% activity. Experiments were performed in triplicates, results are shown mean ± SD; ^∗∗^*P* ≤ 0.01; ^∗∗∗^*P* ≤ 0.001.

The XTT assay was employed to establish anti-biofilm potential of as-synthesized ZnO-NRs-and DAS mediated therapy. The results exhibited the dose dependent antibiofilm effect of both ZnO-NRs and ZnO-NRs-DAS emulsion (**Figure 6**). Further the ZnO-NRs in combination with DAS was able to inhibit the biofilm in more effective way as compared to the individual treatment with ZnO-NRs and DAS alone (*p*-value < 0.01 (^∗∗^)) [Data not shown for MRSA ATCC 43300]. The observed enhanced antibacterial activity could be attributed to the shielding of the ZnO-NRs with DAS that allows the slow release of zinc ions. The prolonged release of Zinc ions make it more potent antibacterial agent. On the other hand DAS inhibits the biofilm formation through Quorum sensing blocking (Cai et al., 2007; Li et al., 2015) as described by different scientific reports. The chemo-resistance of biofilm forming bacteria involves the matrix, which represents a physical and chemical barrier to the antibiotics (Cutler and Wilson, 2004; Wang et al., 2006; Packiavathy et al., 2013). A combination of ZnO-NRs and DAS exerts an antibacterial action by affecting biofilm formation tendancy of drug resistant bacteria. It is envisioned that the combination therapy using ZnO-NRs and DAS enhances both anti-microbial and antibiofilm activities as compared to single therapy by DAS or ZnO-NRs.

**FIGURE 7:**
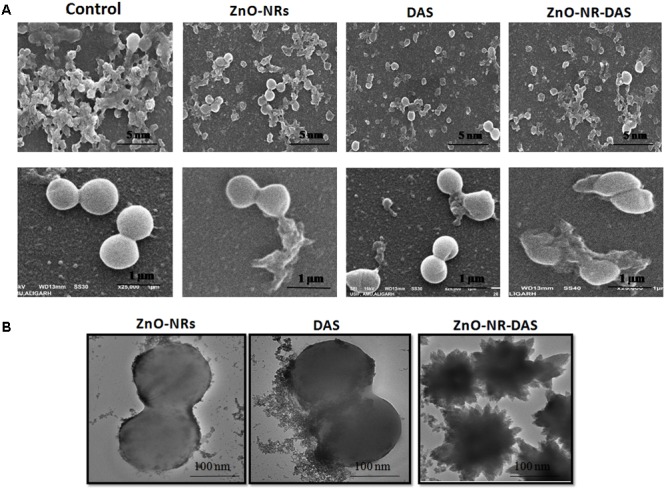
Effect of ZnO-NR-DAS combination on exterior morphology of MRSA as revealed by electron microscopy **(A)** SEM micrograph depicting interaction of various ZnO-NRs with MRSA ATCC 43300strain at different resolutions. **(B)** TEM micrograph showing interaction of various ZnO NRs formulations with MRSA ATCC BAA 1708 strain.

Ultra-structure of *S. aureus* was probed by employing electron microscopy, wherein morphological changes incurred in bacterial cells were deduced by SEM. The control untreated MRSA ATCC BAA 1708 showed round and smooth morphology with identical shape. The exposure to ZnO-NRs-DAS formulations resulted in abrupt change in morphology with damaged surface (**Figure 7A**). Further, TEM analysis suggests lysis of bacterial cells with release of cytosolic content upon their exposure to ZnO-NRs-DAS formulation. (**Figure 7B**) (Cai et al., 2007; Li et al., 2015).

### Protein Profile of MRSA Upon Treatment With ZnO-NRs-DAS Combination

**FIGURE 8:**
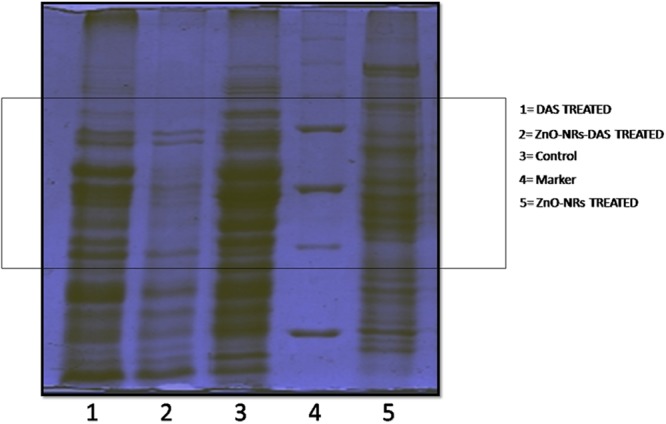
SDS–PAGE profile of MRSA whole proteins upon treatment with ZnO-NRs-DAS. Various lanes corresponding to ZnO-NR-DAS treated MRSA: MRSA ATCC BAA 1708, Lane-1: protein marker; Lane-2: untreated control; Lane-3: ZnO-NRs treatment and Lane-4: DAS treatment; Lane-5: ZnO-NRs-DAS emulsion treatment.

To further investigate the probable antibacterial mechanisms of ZnO-NRs-DAS emulsion, the whole protein profile of treated MRSA was analyzed. The results demonstrated that the membrane integrity of the treated bacteria was compromised. Lane 1, 2, 3, and 4 corresponds to molecular weight marker, control, DAS, ZnO-NRs and ZnO-NRs-DAS emulsion treated MRSA ATCC BAA 1708 bacterial cell lysate, respectively. The major breakdown of proteins observed in the ZnO-NRs-DAS combination as characterized by the absence of large group of bands (**Figure 8**). The protein profiles of bacteria treated with ZnO-NRs and DAS differed from those of the control (untreated) in each case. Compared with the untreated cells, the bands corresponding to 30 and 26 kDa were degraded in *S. aureus* samples. The membrane-associated protein TcaA (TcaA: 52 kDa) has been reported to play a critical role in the synthesis of cell wall in *S. aureus* (Fournier and Hooper, 2000). The degradation and destruction of the critical functional proteins perhaps led to inactivation of the microorganism.

### Antibacterial Potential of ZnO-NRs-DAS Combination Against *S. aureus* Skin Infection

**FIGURE 9:**
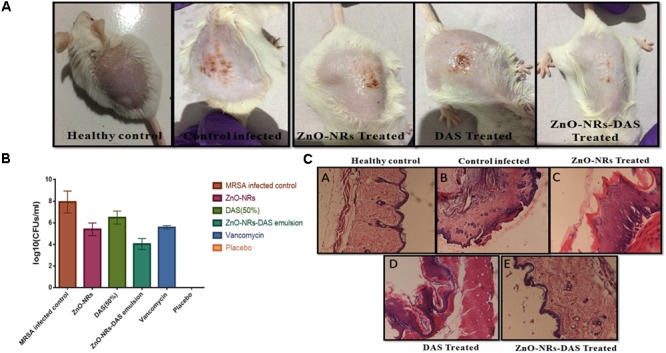
Effect of ZnO-NR-DAS combination on MRSA induced skin infection in Balb/C mice **(A)** Efficacy of ZnO-NR-DAS combination against MRSA induced skin infection. **(B)** Mice were infected topically with MRSA ATCC BAA 1708. The infected mice were treated with ZnO-NR and DAS based formulations. Mice inoculated with PBS alone were used as a healthy control. On day 10 post treatment, small pieces corresponding to skin lesions were cut, homogenized and bacterial count was determined employing CFU assay. **(C)** On day 10, biopsy specimens were fixed in 4% neutral buffered formalin and embedded in parafilm. The specimens were stained with hematoxylin and eosin to perform histopathological study.

Taking cue-from *in vitro* data that suggest strong antibacterial potential of ZnO-NRs-DAS combination against both MRSA ATCC 43300 as well as ATCC BAA-1708 isolates, next we assessed its potential to treat acute bacterial skin infection in animal model (Cutler and Wilson, 2004; Sharifi-Rad et al., 2014). The exposure with MRSA resulted in cutaneous bacterial infections that was characterized by reddening of the skin that eventually resulted in disruption of localized skin (**Figure 9A**). The bacterial load in the skin was assessed by allowing the enumeration of bacteria present in the given specimen. In accordance to the previous results from our lab where we employed Zinc oxide nanoparticles for the treatment of *S. aureus* based skin infection (Rauf et al., 2017), the ZnO-NRs successfully eliminated skin infection (**Figure 9B**). Interestingly, Supplementing ZnO-NR with DAS resulted in tremendous increase in its potential to reduce bacterial burden (∼ 60% reduction) when compared to untreated control group (*P* < 0.005).

**FIGURE 10:**
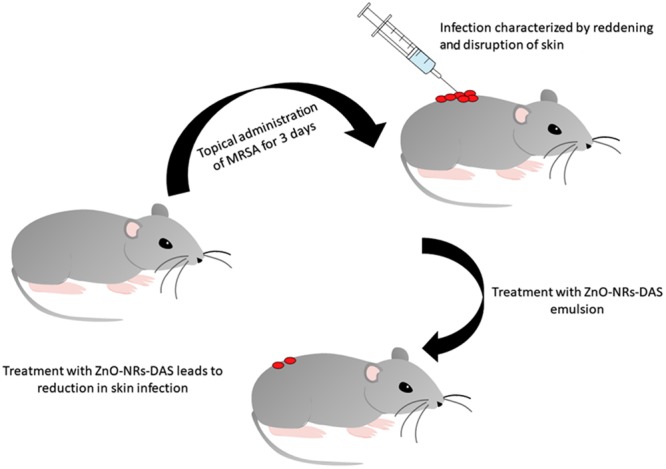
Schematic illustration showing potential of ZnO-NRs-DAS emulsion in treatment of skin infection in mouse model.

### Histopathological Analysis

We examined the skin architecture, bacterial burden and the incurred inflammatory changes in infected mice to assess the efficacy of various ZnO-NRs formulations. Lesions on the skin were taken out aseptically on day 10 post-infection and stained employing hematoxylin and eosin (HE). The control healthy group showed healthy skin histology with a normal intact epidermal layer, whereas MRSA infected skin showed thinning of the epidermal layer with disruption along with the presence of a large number of inflammatory cells. Treatment with ZnO-NRs-DAS resulted in healing of epidermal layer with minimal skin damage. Overall, the skin acquired normal architecture upon treatment with ZnO-NRs-DAS treatment (**Figure 9C**).

## Conclusion

In the present study, we have employed egg white mediated biomimetic approach for the synthesis of ZnO-NRs. The as-synthesized ZnO-NRs were characterized employing UV and FT-IR spectroscopy, the surface structure, size dimensions and crystalline nature of as-synthesized ZnO-NRs was assessed employing XRD and electron microscopic techniques. The as-synthesized ZnO-NRs showed strong antimicrobial activity against both sensitive as well as resistant SA strains. The antimicrobial as well as antibiofilm potential of ZnO-NRs was found to be further enhanced when used in combination with DAS. The as-synthesized ZnO-NRs-DAS combination demonstrated strong potential to inhibit both sensitive as well as resistant *S. aureus* pathogen. The combination therapy was also successful in suppression of *S. aureus* mediated skin infection in BALB/c mice. It can be speculated that the ZnO-NR and DAS combination is likely to kill other drug resistant microbes as well.

### Statistical Analysis

Results were expressed as the mean ± SD and data were analyzed by means of both one way analysis of variance (ANOVA) and two-way ANOVA to assess the differences among various groups. Statistical calculations were performed with the help of Graph-Pad prism version 6.0, Graphpad software Inc., San Diego, CA, United States. Significance was indicated as ^∗∗∗^ for *P* ≤ 0.001; ^∗∗^ for *P* ≤ 0.01 and ^∗^ for *P* ≤ 0.05. Student’s *t*-test was used to measure biochemical differences between treatment groups and differences with *P* ≤ 0.05 considered to be significant.

## Author Contributions

MR, MO, and SZ conceived and designed the experiments. MR, HA, KD, and SP performed the experiments. MO and MR analyzed the data. MA performed the histopathological analysis. MO and SZ contributed reagents, materials and analysis tools. MR and MO wrote the first draft of the manuscript.

## Conflict of Interest Statement

The authors declare that the research was conducted in the absence of any commercial or financial relationships that could be construed as a potential conflict of interest. The handling Editor declared a shared affiliation, though no other collaboration, with the authors.

## Footnotes

1 https://www.nutrasilver.com/mrsa
