# Supplementary material for: Synergistic Effect of Diallyl Sulfide With Zinc Oxide Nanorods: A Novel and Effective Approach for Treatment of Acute Dermatitis in Model Animals
Source: Front Microbiol. 2018 Apr 18;9:586. doi: 10.3389/fmicb.2018.00586 (PMC5915547; doi:10.3389/fmicb.2018.00586)
Supplement: Supplementary file 1 [file Data_Sheet_1.docx]

**Methodology**

**UV-Visible spectroscopy**

Ultraviolet-Visible (UV) spectra of as-synthesized ZnO-NRs was recorded on a double beam spectrophotometer (Shimadzu) operated at a resolution of 1 nm in the range A_200_ to A_700_ nm. [**Zare, Elham, et al 2017, Prasad, Virendra, et al 2006**]

**Surface properties of as-synthesized ZnO-NRs employing Transmission and Scanning Electron Microscopy**

The as-synthesized nanorods were characterized employing TEM and SEM analysis following method as described elsewhere [**Alam, Umair, et al 2018,** **MA Rauf et al 2017**]. The sample was prepared by placing a drop of reaction product over gold coated negative grid, allowing the solution to evaporate. TEM was performed on JEOL model electron microscope. The microscope was operated at an accelerating voltage of 1000kv.For surface morphological analysis scanning electron microscopy SEM was performed (JSM67500F, JEOL model).

**X-ray diffraction analysis**

XRD analysis of as-synthesized ZnO-NRs was performed in the 2θ range of 20-80° (RigakuMiniflex II) with Cu K_α_ radiations (λ = 1.5406 Å) operating at a voltage of 30 kV and current of 15 mA. All the diffraction patterns were recorded as step-scans. To run a step-scan, powdered ZnO-NRs were mounted followed by fixing the tube voltage and current, and feeding the following parameters: starting 2-theta angle, step-size (typically 0.005 degrees), count time per step (typically 0.05-1 second) and ending 2-theta angle [**Wang, Zhong Lin et al 2004**, **Wu, Chia Ling, et al 2006**].

**Determination of particle size**

DLS (Dynamic light Scattering) measurement was employed to determine the average size and size dispersal of the ZnO-NRs using a Nanosizer 90ZS (Malvern Instruments, UK).The intensity of scattered light was sensed at 90º to the incident beam. The ZnO-NRs (2 mg/ml) were filtered through 0.22 µm syringe filters directly into a 12 µl quartz cuvette. The data analysis was performed in default mode. The measured size was presented as the average value of 20 runs, with triplicate measurements within each run. [**Prasad, Virendra, et al 2006**, **MA Rauf et al 2017]**

**Determination of minimum inhibitory concentration (MIC)**

Minimum inhibitory concentration (MIC) is the lowest concentration of an antimicrobial agent that may prevent the visible growth of microorganisms after stipulated time period. MIC is considered as a significant parameter in diagnostic laboratories to ascertain sensitivity of a microorganism against a particular antimicrobial agent. The MIC value of as-synthesized ZnO-NRs was determined by microdilution method against MRSA ATCC 43300 and MRSA ATCC BAA-1708 strains. The MIC values were estimated on the basis of viability test performed in 96-well microdilution plates according to the previously developed protocols **[Ferraro, Mary Jane et al 2003**].

**Antibacterial properties of as-synthesized ZnO-NRs as determined by Agar diffusion assay**

The overnight grown bacterial culture was centrifuged at 5000 g for 5 minutes. The bacterial pellet was washed with sterile PBS of 7.4 pH and again resuspended in HEPES buffer. An aliquot (100 μl) of the suspended culture was spread evenly on LB agar plate with a sterile glass spreader and incubated at 37°C. Subsequently the wells were bored using gel borer. After 1 hr of incubation period various test samples were dispensed in formed wells. The plate was exposed to increasing concentration of ZnO-NRs from 10mg/ml stock solution. Zone of inhibition was determined estimating bacteria free zone around the well after 24 hrs [**Nature protocols 2007, MA Rauf et al 2017**].The experimental procedure was performed under sterile conditions using level-2 bio-safety hoods. The experiments were performed in triplicate. The mean value was calculated and compared with the reference drug vancomycin as a control.

**Determining the growth curves of bacterial cells exposed to Different formulations**

The growth curves of MRSA after exposure to different Zinc oxide and DAS formulations were plotted with the optical density (OD) versus time. Both MRSA (0.2 mL, ∼10^8^ CFU mL^−1^) was inoculated in 10mL of a fresh LB medium supplemented with different formulations at their respective MICs concentrations. The mixtures were then incubated in a rotary shaker at 150 rpm at 37 °C. The growth was monitored at an interval of every 2 hour by measuring the increase of the OD at 600 nm using an UV−vis spectrophotometer (U-3010, Hitachi, Japan). All of the experiments were performed in triplicate, and the results are presented as mean ± standard deviation.

**CFU assessment to evaluate bacterial susceptibility against as-synthesized ZnO-NRs and DAS emulsion**

The overnight grown culture of various bacterial strains were sub-distributed into 6 culture tubes (adjusted density to 10^6^-10^7^ cells/ml).Further, 100 µl aliquot of ZnO-NRs solution from stock solution of 10mg/ml followed by NRs-DAS emulsion and DAS alone and final stock of vancomycin solution (100 µg/ml), Negative control (without culture and without formulation) and Positive control (culture+100 µl PBS) was dispensed to corresponding tubes and allowed to incubate for further 4 hours at 37 °C. Thereafter, 100µL suspension from each of the treated and control group tubes was plated in duplicate up to two different dilutions (1:1, 1:10) on to the TSB congo red agar plates and incubated further at 37°C. After 24 hrs of incubation at 37 °C, resultant colony forming units (CFU) at different dilutions were counted, averaged and expressed as log10 CFU/ml and the counts from two independent experiments were averaged [**Tavaf, Zohreh, et al. 2017**, **Shahverdi, Ahmad R., et al. 2007**]

**REFERENCES**

Alam, Umair, et al. "Synthesis of iron and copper cluster-grafted zinc oxide nanorod with enhanced visible-light-induced photocatalytic activity." *Journal of colloid and interface science*509 (2018): 68-72.

Ferraro, Mary Jane. "An NCCLS global consensus standard, Methods for dilution antimicrobial susceptibility tests for bacteria that grow aerobically." *Approved Standard M7-A6*(2003).

Prasad, Virendra, et al. "Spectroscopic characterization of zinc oxide nanorods synthesized by solid-state reaction." *Spectrochimica Acta Part A: Molecular and Biomolecular Spectroscopy* 65.1 (2006): 173-178.

Rauf, Mohd Ahmar, et al. "Biomimetically synthesized ZnO nanoparticles attain potent antibacterial activity against less susceptible S. aureus skin infection in experimental animals." *RSC Advances* 7.58 (2017): 36361-36373.

Shahverdi, Ahmad R., et al. "Synthesis and effect of silver nanoparticles on the antibacterial activity of different antibiotics against Staphylococcus aureus and Escherichia coli." *Nanomedicine: Nanotechnology, Biology and Medicine*3.2 (2007): 168-171.

Tavaf, Zohreh, et al. "Evaluation of antibacterial, antibofilm and antioxidant activities of synthesized silver nanoparticles (AgNPs) and casein peptide fragments against Streptococcus mutans." *European Journal of Integrative Medicine* 12 (2017): 163-171.

Wang, Zhong Lin. "Zinc oxide nanostructures: growth, properties and applications." *Journal of physics: condensed matter* 16.25 (2004): R829.

Wiegand, I., Hilpert, K., and Hancock, R. E. W. (2008). Agar and broth dilution methods to determine the minimal inhibitory concentration (MIC) of antimicrobial substances. Nat. Protoc. 3, 163–175.

Wu, Chia Ling, et al. "Growth and characterization of chemical-vapor-deposited zinc oxide nanorods." *Thin solid films* 498.1-2 (2006): 137-141.

Zare, Elham, et al. "Simple biosynthesis of zinc oxide nanoparticles using nature's source, and it's in vitro bio-activity." *Journal of Molecular Structure* 1146 (2017): 96-103.
